# Why are the Pakistani maternal, fetal and newborn outcomes so poor compared to other low and middle-income countries?

**DOI:** 10.1186/s12978-020-01023-5

**Published:** 2020-12-17

**Authors:** Aleha Aziz, Sarah Saleem, Tracy L. Nolen, Nousheen Akber Pradhan, Elizabeth M. McClure, Saleem Jessani, Ana L. Garces, Patricia L. Hibberd, Janet L. Moore, Shivaprasad S. Goudar, Sangappa M. Dhaded, Fabian Esamai, Constance Tenge, Archana B. Patel, Elwyn Chomba, Musaku Mwenechanya, Carl L. Bose, Edward A. Liechty, Nancy F. Krebs, Richard J. Derman, Waldemar A. Carlo, Antoinette Tshefu, Marion Koso-Thomas, Sameen Siddiqi, Robert L. Goldenberg

**Affiliations:** 1 Department of Obstetrics and Gynecology, Columbia University School of Medicine, New York, NY USA; 2 Aga Khan University, Karachi, Pakistan; 3 RTI International, Durham, NC USA; 4 Instituto de Nutrición de Centroamérica y Panamá, Guatemala, Guatemala; 5 School of Public Health, Boston University, Boston, MA USA; 6 J N Medical College, KLE Academy Higher Education and Research, Belagavi, Karnataka India; 7 Moi University School of Medicine, Eldoret, Kenya; 8 Lata Medical Research Foundation, Nagpur, India; 9 University Teaching Hospital, Lusaka, Zambia; 10 University of North Carolina at Chapel Hill, Chapel Hill, NC USA; 11 Indiana School of Medicine, University of Indiana, Indianapolis, IN USA; 12 University of Colorado School of Medicine, Denver, CO USA; 13 Thomas Jefferson University, Philadelphia, PA USA; 14 University of Alabama at Birmingham, Birmingham, AL USA; 15 Kinshasa School of Public Health, Kinshasa, Democratic Republic of the Congo; 16 Eunice Kennedy Shriver National Institute of Child Health and Human Development, Bethesda, MD USA

**Keywords:** Pakistan, Pregnancy outcomes, Maternal mortality, Stillbirth, Neonatal mortality, Risk factors, Global network

## Abstract

**Background:**

Pakistan has among the poorest pregnancy outcomes worldwide, significantly worse than many other low-resource countries. The reasons for these differences are not clear. In this study, we compared pregnancy outcomes in Pakistan to other low-resource countries and explored factors that might help explain these differences.

**Methods:**

The Global Network (GN) Maternal Newborn Health Registry (MNHR) is a prospective, population-based observational study that includes all pregnant women and their pregnancy outcomes in defined geographic communities in six low-middle income countries (India, Pakistan, Democratic Republic of Congo, Guatemala, Kenya, Zambia). Study staff enroll women in early pregnancy and follow-up soon after delivery and at 42 days to ascertain delivery, neonatal, and maternal outcomes. We analyzed the maternal mortality ratios (MMR), neonatal mortality rates (NMR), stillbirth rates, and potential explanatory factors from 2010 to 2018 across the GN sites.

**Results:**

From 2010 to 2018, there were 91,076 births in Pakistan and 456,276 births in the other GN sites combined. The MMR in Pakistan was 319 per 100,000 live births compared to an average of 124 in the other sites, while the Pakistan NMR was 49.4 per 1,000 live births compared to 20.4 in the other sites. The stillbirth rate in Pakistan was 53.5 per 1000 births compared to 23.2 for the other sites. Preterm birth and low birthweight rates were also substantially higher than the other sites combined. Within weight ranges, the Pakistani site generally had significantly higher rates of stillbirth and neonatal mortality than the other sites combined, with differences increasing as birthweights increased. By nearly every measure, medical care for pregnant women and their newborns in the Pakistan sites was worse than at the other sites combined.

**Conclusion:**

The Pakistani pregnancy outcomes are much worse than those in the other GN sites. Reasons for these poorer outcomes likely include that the Pakistani sites' reproductive-aged women are largely poorly educated, undernourished, anemic, and deliver a high percentage of preterm and low-birthweight babies in settings of often inadequate maternal and newborn care. By addressing the issues highlighted in this paper there appears to be substantial room for improvements in Pakistan’s pregnancy outcomes.

## Background

The United Nations’ establishment of Millennium Development Goals (MDGs) 4 and 5 – to improve child and maternal health, respectively, brought high maternal and neonatal mortality to the forefront of the global stage. These goals included a three-quarters reduction in the 1990 maternal mortality ratio (MMR) and a two-thirds reduction in the 1990 under-5 mortality rate, both to be achieved by 2015. Knowledge about rates and trends in maternal and neonatal mortality, as well as for stillbirths, can identify particular sub-populations that may be at higher risk for death, and inspire strategies to reduce this risk.

While Pakistan has shown a decrease in its MMR and neonatal mortality rate (NMR) since 1990, there has been less improvement in these outcomes as compared to other south Asian countries [1–3]. Stillbirth rates have rarely been addressed and remain under-reported in Pakistan, but are approximately equal in number to the neonatal deaths. In 2015, there were about 5,500,000 births in Pakistan [4]. The MMR in 2015 in Pakistan was reported as 178 deaths/100,000 live births, decreased by 58.7% since 1990, when the rate was 431 deaths/100,000 live births [1]. However, in rural areas of Pakistan, the MMR in 2007 was reported as almost twice that figure, at 319 deaths/100,000 live births, with wide variation between provinces—227 in Punjab vs. 785 in Baluchistan [5]. Because these data are derived from a poorly functioning vital statistics system or estimates based on sampling, they likely under-report deaths.

Pakistan’s overall NMR in 2015 was reported as 44 deaths per 1000 live births. However, in rural areas, the NMR was reported to be 62 deaths per 1,000 live births, while for the richest households, the NMR was reported as 34 deaths per 1000 live births [6, 7]. The stillbirth rate in 2015 was reported at 43 per 1000 total births [7]. In the Pakistan 2017–2018 Demographic Health Survey (PDHS), the NMR for the five years preceding the survey was 42 deaths per 1000 live births [8]. Again, because of the lack of a functioning vital statistics system, these numbers are at best estimates and the number of deaths are likely to be underreported.

Pakistan consistently lagged in achieving the health-related MDGs 4 and 5 for reducing maternal and child mortality. This led the government to launch the National Maternal, Neonatal, and Child Health (MNCH) Programme in 2007, to improve maternal and child health outcomes. This program concentrated on two main areas: (1) providing emergency obstetric services and community midwives, and (2) promoting institutional deliveries and skilled birth attendance. Lady health workers (LHWs) provided obstetric and newborn services, including primary health care through home visits in rural areas. Unfortunately, despite these efforts, the MDG targets were not achieved [8]. As a signatory of the newer Sustainable Development Goals (SDGs) 2015–2030, the government of Pakistan developed a monitoring and evaluation mechanism for the National Health Vision 2016–2025. The resulting framework is coordinated with the Planning Commission of Pakistan for SDG reporting, as well as with other stakeholders to ensure an inclusive and wide reach [8]. The SDG 3—to ensure healthy lives and promote well-being at all ages—includes bringing the global NMR to as low as 12 deaths per 1000 live births and the MMR to less than 70 deaths per 100,000 live births by 2030 [9].

Countries such as Rwanda, Iran, Bhutan, Cambodia, the Lao People’s Democratic Republic, Mongolia, and Timor-Leste are categorized as having “achieved MDG 5A” based on MMR reduction estimates indicating a downward trend of at least 75% between 1990 and 2015 [1]. On the other hand, in Pakistan, the overall MMR decrease did not achieve that goal [10]. Furthermore, in a recent report by UNICEF in 2018, Pakistan was listed as the riskiest place in the world to be born as measured by its NMR in 2016—higher than that of Central African Republic, Afghanistan, and Somalia. One in 22 babies born in Pakistan in 2016 died before the end of the first month of life [2].

The Global Network (GN) for Women’s and Children’s Health Research’s Maternal Newborn Health Registry (MNHR) provides a unique opportunity to compare pregnancy outcomes and putative explanatory factors for adverse outcomes at a surveillance site in Pakistan with sites in six other countries. The purpose of this paper is to compare the MMR, rates of stillbirth, and NMR as well as factors that might help to explain the differences between the site in Pakistan and six other sites in the MNHR, including two sites in India, as well as sites in Kenya, Zambia, the Democratic Republic of the Congo (DRC), and Guatemala.

## Methods

Data for this study were collected by the *Eunice Kennedy Shriver* National Institute of Child Health and Human Development (NICHD)’s Global Network for Women and Children’s Health Research. The GN’s MNHR, established in 2008, is a multi-site, prospective, ongoing active surveillance system to track pregnancies and births in GN sites (seven at the time of this study): Chimaltenango, Guatemala; Nagpur and Karnataka Districts, India; western Kenya; Thatta District, Pakistan; areas near Lusaka, Zambia; North and South Ubangi Province, DRC (since 2014). Each site is composed of clusters that are defined geographic areas generally related to a health center. Approximately 300–500 births per year occur in each cluster. The number of clusters has varied in each site over time. Descriptions of the MNHR and its history as well as the individual sites are included elsewhere [11]. In general, the data elements were collected consistently throughout the study, although in some cases, a particular data element was added or discontinued. In each of the tables, the number of cases for which we had data for a specific characteristic is listed.

Data on the eligible pregnant women and their babies were obtained at three time points. The first visit, at enrollment, ideally occurred by week 20 of gestation and data used to date the pregnancy were collected, as well as the mother’s age, level of schooling, parity, date of delivery and status of last child born. Hemoglobin values were collected at greater frequency as the study progressed, especially in India and Pakistan. In the last 2 years of this study, data on socioeconomic status have also been collected. The second visit occurs within 48 hours of delivery and information collected includes the number of antenatal care (ANC) visits, complications during pregnancy, details of labor and delivery, including place, mode of delivery, provider and practices, birth weight, status of the mother and newborn following delivery, referrals, and treatment provided to the mother and newborn. Interval maternal and newborn health and vital status are assessed at a third visit on day 42 after birth. The same study protocol and similar operational mechanisms are utilized at all the sites across the GN [12].

Thatta is a predominantly rural district bordering Karachi and Hyderabad, the two largest cities in the province of Sindh. Despite its close proximity to these urban centers, Thatta was ranked 90th of 114 districts in Pakistan on the Human Development Index in 2017 and was in the bottom five among the 24 districts within the province of Sindh [13]. Recent reports from 2016 show that the education sector in Thatta, in particular, lags behind the rest of the country. Thatta has the lowest educational attainment score in the province and is ranked amongst the 15 lowest districts in the country [14]. On the other hand, Thatta does have a large number of health care providers spread throughout the district, and the proportion of women delivering at health care facilities is higher than the national average [15].

The study has been reviewed and approved at all of the involved institutions’ ethics review committees including the review committees at the US institutions that partnered with each of the low and middle-income sites. A Data Monitoring Committee appointed by NICHD reviews the MNHR data on a semiannual basis. All enrolled women provide informed consent to participate in the MNHR.

### Analysis

We compared the data collected in the sites in India, Kenya, Zambia, the DRC and Guatemala with those from Pakistan for the years 2010–2018. For most summaries, because of regional characteristics and outcome similarities, data from the two sites in India were combined as were data from the Zambian and Kenyan sites. The DRC site was considered separately since we only had data from 2014 onward, and the mortality rates were substantially higher than the other two other African sites. The Guatemalan site, as the only Central American site, was also considered separately. Data were entered at each study site with data edits performed prior to transmission to a central data center (RTI International, Durham, NC, USA) where additional data edits were performed. Data were analyzed centrally and statistical analyses performed using SAS v. 9.4.

Descriptive analyses are reported for the delivery and health care characteristics, stratified by region. These characteristics are compared between the Pakistani site and all other sites combined using chi-square tests for categorical characteristics and t tests for continuous measures. We modeled the risk of maternal mortality, stillbirth, and neonatal mortality and calculated point and interval estimates of risk ratios using log binomial models. We used generalized estimating equations to account for correlation of outcomes within clusters to assure appropriately sized p values and confidence intervals. Because mortality trend data through 2013 was presented earlier, and because the DRC only had data available for 2014–2018, for mortality trend analyses, we included only those five years. To evaluate changes in outcomes over time, we modeled year of delivery and tested for trends with an orthogonal polynomial linear contrast.

Hemoglobin levels have been collected in the MNHR since 2008 when available, but only consistently in the Indian sites since about 2014 and only more recently in the Pakistani site. In the other sites, even in recent years, hemoglobin values were available on fewer than half the women. For this reason, our analysis of the hemoglobin data included only those values from India and Pakistan. A more thorough description of the hemoglobin levels in the Indian and Pakistani sites is included in another paper in this supplement [16]. The socioeconomic data were collected in all sites only from 2017 to 2018 and are also described in detail in another paper in this supplement [17]. To provide a sense of where the Pakistani site falls among the sites, only the ranking of socioeconomic status (SES) among the sites is presented here. The inter-delivery interval (IDI)—time from delivery of one live last birth to the delivery of the index birth—is also described in detail in another paper in this supplement [18]. Therefore, data for the percent of women with a short IDI comparing the Pakistan site to all the other sites except Pakistan are presented only in the text.

## Results

**Fig. 1:**
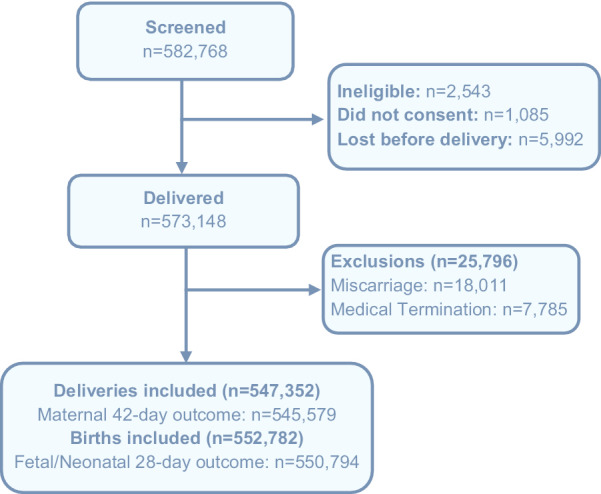
CONSORT diagram

Figure 1 shows the population included and excluded in this study. Over the nine years (2010–2018), data on a total of 91,076 pregnancies in the Pakistan site and 456,276 pregnancies in the six other sites were collected. Across all sites, among those enrolled, follow-up at six weeks after delivery was 99.2% (ranging from 99.0 to 99.8%).

Table 1 shows the number of deliveries as well as the MMR, NMR and stillbirth rates for all sites (grouping the Indian sites together and the Kenyan and Zambian sites together) and then for all sites excluding Pakistan. In this analysis, the Pakistani site had an MMR almost three times that of the other sites combined—although the MMRs for the DRC and Pakistani sites were similar. The stillbirth rates and the NMR in the Pakistani site were more than twice those of the other sites combined.

**Table 1:** The maternal mortality ratios, and stillbirth and neonatal mortality rates for all sites and for all sites except Pakistan 2010 to 2018

Variable	Pakistan	India	Kenya and Zambia	DRC	Guatemala	Total except Pakistan
Deliveries, N	91,076	205,203	136,567	31,604	82,902	456,276
Births, N	92,166	206,816	138,146	32,173	83,481	460,616
42-day maternal mortality ratio, n/N (rate/100,000 live births)	278/87,172 (319)	237/201,722 (117)	142/135,300 (105)	98/30,894 (317)	79/81,861 (97)	556/449,777 (124)
Stillbirth, n/N (rate/1000 births)	4923/92,095 (53.5)	5019/206,741 (24.3)	2807/138,107 (20.3)	1249/32,143 (38.9)	1610/83,471 (19.3)	10,685/460,462 (23.2)
28-day neonatal mortality, n/N (rate/1000 live births)	4269/86,455 (49.4)	4489/201,550 (22.3)	1967/134,632 (14.6)	742/30,871 (24.0)	1936/81,678 (23.7)	9134/448,731 (20.4)

Table 2 shows the MMR, stillbirth and NMR rates for each site, or groups of sites as described above, and for all sites but Pakistan for the individual years 2014–2018. While the MMR was significantly higher for the Pakistani site versus the other sites combined (p < 0.0001), the MMR did not significantly change between 2014 and 2018 for either the Pakistani site nor for the other sites combined (p = 0.3229 and 0.9012 respectively). Rates of stillbirths were also significantly higher in the Pakistani site versus the other sites combined (p < 0.0001). The Pakistani site did see a significant reduction in the stillbirth rate between 2014 and 2018 (p = 0.0009) that appears to be linear (p = 0.0011) while the other sites overall did not have a significant reduction (p = 0.2063). The NMR was again significantly higher in the Pakistan site versus the other sites combined (p < 0.0001); however, the Pakistani site did not have a significant reduction between 2014 and 2018 (p = 0.2436) while the other sites combined did have a reduction (p = 0.0005) that appears to be linear (p = 0.0026).

**Table 2:** Trends in the maternal mortality ratio, stillbirth and neonatal mortality rates 2014–2018 for ongoing clusters and the percent change from 2014 to 2018 for the Pakistan site and the Indian, African and Guatemalan sites and for all sites but Pakistan

Variable	Pakistan	India	Kenya and Zambia	DRC	Guatemala	Total except Pakistan
Maternal mortality < 42 days, n/N (rate/100,000 deliveries)						
2014	19/6810 (279)	19/15,720 (121)	14/14,351 (98)	20/6062 (330)	7/6965 (101)	60/43,098 (139)
2015	20/6360 (314)	12/16,113 (74)	14/14,802 (95)	22/6050 (364)	8/7317 (109)	56/44,282 (126)
2016	20/6057 (330)	15/14,951 (100)	9/14,831 (61)	10/6312 (158)	10/7888 (127)	44/43,982 (100)
2017	28/5932 (472)	17/13,675 (124)	18/14,551 (124)	22/6709 (328)	11/7410 (148)	68/42,345 (161)
2018	20/5735 (349)	15/12,238 (123)	11/14,289 (77)	24/6442 (373)	6/6846 (88)	56/39,815 (141)
Comparisons	RR estimate	RR 95% CI	RR P value	Trend P value		
Pakistan vs. total except Pakistan	2.56	2.05, 3.20	< 0.0001			
Pakistan: 2018 vs. 2014	1.27	0.79, 2.02	0.3229	0.1134		
Total except Pakistan: 2018 vs. 2014	1.02	0.73, 1.42	0.9012	0.5040		
Stillbirth, n/N (rate/1000 births)						
2014	379/6886 (55.0)	381/15,834 (24.1)	290/14,571 (19.9)	223/6168 (36.2)	119/7038 (16.9)	1,013/43,611 (23.2)
2015	342/6446 (53.1)	396/16,261 (24.4)	277/15,044 (18.4)	257/6165 (41.7)	172/7384 (23.3)	1,102/44,854 (24.6)
2016	302/6141 (49.2)	356/15,104 (23.6)	310/15,050 (20.6)	268/6429 (41.7)	177/7939 (22.3)	1,111/44,522 (25.0)
2017	284/5996 (47.4)	297/13,768 (21.6)	320/14,836 (21.6)	236/6830 (34.6)	159/7446 (21.4)	1,012/42,880 (23.6)
2018	257/5794 (44.4)	216/12,339 (17.5)	247/14,554 (17.0)	265/6551 (40.5)	139/6892 (20.2)	867/40,336 (21.5)
Comparisons	RR estimate	RR 95% CI	RR P value	Trend P value		
Pakistan vs. total except Pakistan	2.03	1.79, 2.30	< 0.0001			
Pakistan: 2018 vs. 2014	0.80	0.70, 0.91	0.0009	0.0011		
Total except Pakistan: 2018 vs. 2014	0.93	0.82, 1.04	0.2063	0.1463		
28-day neonatal mortality, n/N (rate/1000 live births)						
2014	305/6498 (46.9)	343/15,451 (22.2)	182/14,221 (12.8)	152/5933 (25.6)	210/6900 (30.4)	887/42,505 (20.9)
2015	336/6098 (55.1)	314/15,863 (19.8)	190/14,699 (12.9)	143/5904 (24.2)	202/7207 (28.0)	849/43,673 (19.4)
2016	295/5828 (50.6)	303/14,737 (20.6)	210/14,685 (14.3)	145/6159 (23.5)	224/7758 (28.9)	882/43,339 (20.4)
2017	286/5701 (50.2)	262/13,468 (19.5)	203/14,397 (14.1)	171/6593 (25.9)	163/7285 (22.4)	799/41,743 (19.1)
2018	283/5532 (51.2)	211/12,120 (17.4)	156/14,183 (11.0)	131/6282 (20.9)	160/6749 (23.7)	658/39,334 (16.7)
Comparisons	RR estimate	RR 95% CI	RR P value	Trend P value		
Pakistan vs. total except Pakistan	2.64	2.41,2.90	< 0.0001			
Pakistan: 2018 vs. 2014	1.10	0.94, 1.29	0.2436	0.5819		
Total except Pakistan: 2018 vs. 2014	0.81	0.71, 0.91	0.0005	0.0026		

We next examined maternal characteristics that could help explain the large differences in maternal, fetal and neonatal outcomes between the Pakistani site and the sites in India, Kenya and Zambia, the DRC and Guatemala, and for all sites but Pakistan combined (Table 3). Education and parity were notably different in the Pakistani site compared to the other sites combined. More than 80% of the pregnant women in the Pakistani site had no formal education compared to a combined rate of 12.4% across the other sites. While 95% of pregnant women in the Indian sites had a parity of two or less, around 50% of pregnant women in the Pakistani and DRC sites had a parity of three or more. Furthermore, the rate of parity more than four in the Pakistani site was three times greater than that of the other sites combined. Additionally, both Pakistani and Indian women had a lower body mass index (BMI) than women in the other sites. The maternal age tended to be different between the Pakistani and the other sites as well. In the Pakistani site, the percentage of women less than 20 years old at delivery was more than 50% lower than the proportion in the other sites combined.

**Table 3:** Maternal and demographic characteristics for women in the Pakistani site and the Indian, African and Guatemalan sites and for all sites but Pakistan

Variable	Pakistan	India	Kenya and Zambia	DRC	Guatemala	Total except Pakistan
Deliveries, N	91,076	205,203	136,567	31,604	82,902	456,276
Maternal age, N (%)	90,900	205,097	135,890	31,573	82,886	455,446
< 20	3716 (4.1)	14,393 (7.0)	31,666 (23.3)	6047 (19.2)	13,907 (16.8)	66,013 (14.5)
20–35	82,204 (90.4)	190,088 (92.7)	96,060 (70.7)	22,759 (72.1)	60,282 (72.7)	369,189 (81.1)
> 35	4980 (5.5)	616 (0.3)	8164 (6.0)	2767 (8.8)	8697 (10.5)	20,244 (4.4)
Maternal education, N (%)	90,868	204,508	135,779	31,603	82,891	454,781
No formal education	75,064 (82.6)	24,662 (12.1)	7,120 (5.2)	12,037 (38.1)	12,487 (15.1)	56,306 (12.4)
Primary	7183 (7.9)	41,026 (20.1)	52,893 (39.0)	13,945 (44.1)	45,360 (54.7)	153,224 (33.7)
Secondary	6339 (7.0)	113,202 (55.4)	70,155 (51.7)	5549 (17.6)	21,183 (25.6)	210,089 (46.2)
University+	2282 (2.5)	25,618 (12.5)	5611 (4.1)	72 (0.2)	3861 (4.7)	35,162 (7.7)
Parity, N (%)	88,759	204,669	135,939	31,603	82,896	455,107
0	16,902 (19.0)	89,947 (43.9)	39,444 (29.0)	5913 (18.7)	23,995 (28.9)	159,299 (35.0)
1–2	29,772 (33.5)	104,544 (51.1)	52,557 (38.7)	9835 (31.1)	31,875 (38.5)	198,811 (43.7)
3–4	20,089 (22.6)	9395 (4.6)	28,428 (20.9)	8573 (27.1)	13,849 (16.7)	60,245 (13.2)
> 4	21,996 (24.8)	783 (0.4)	15,510 (11.4)	7282 (23.0)	13,177 (15.9)	36,752 (8.1)
Mean (std)	3.0 (2.7)	0.8 (0.9)	1.9 (2.0)	2.8 (2.3)	2.2 (2.4)	1.5 (1.9)
Maternal weight, Mean (n, std)	49.9 (90,737, 9.5)	46.2 (200,839, 7.0)	60.1 (120,118, 9.2)	52.9 (31,602, 7.1)	56.8 (70,098, 9.4)	52.4 (422,657, 10.2)
Maternal BMI, Mean (n, std)	20.9 (90,702, 3.7)	20.0 (199,444, 2.9)	23.6 (77,245, 3.6)	21.5 (31,600, 2.4)	26.2 (64,595, 4.0)	22.0 (372,884, 4.0)
Antenatal care visits, N (%)	69,544	147,626	104,912	31,557	73,317	357,412
0	5177 (7.4)	176 (0.1)	838 (0.8)	1235 (3.9)	2601 (3.5)	4850 (1.4)
1–2	27,628 (39.7)	9078 (6.1)	19,999 (19.1)	6574 (20.8)	10,375 (14.2)	46,026 (12.9)
≥ 3	36,739 (52.8)	138,372 (93.7)	84,075 (80.1)	23,748 (75.3)	60,341 (82.3)	306,536 (85.8)
Tetanus toxoid vaccine, n/N (%)	46,629/90,992 (51.2)	204,674/205,094 (99.8)	125,115/136,555 (91.6)	27,498/31,595 (87.0)	60,146/82,672 (72.8)	417,433/455,916 (91.6)
Vitamins/calcium/iron, n/N (%)	66,389/90,984 (73.0)	203,323/204,901 (99.2)	132,669/136,561 (97.1)	29,614/31,593 (93.7)	78,273/82,881 (94.4)	443,879/455,936 (97.4)

Characteristics compared between the Pakistan site and all other sites combined using chi-square tests for categorical factors and t-tests for continuous measures. All p-values were < 0.0001

Other factors that could help explain the poor maternal, fetal and neonatal outcomes in the Pakistani site include less use of ANC among pregnant women, with only 52.8% Pakistani women attending three or more ANC visits, as compared to 85.8% with three or more visits in the remaining sites. In the Pakistani site, 7.4% of pregnant women did not experience any ANC, compared to 1.4% for the other sites combined. Women in the Pakistani site were less likely to receive immunization with tetanus toxoid or have prenatal vitamin/calcium/iron supplementation than women at any of the other sites.

There were additional variables considered. In a GN study by Ali et al., published in this supplement, focusing on hemoglobin values in pregnancy in Pakistan and India, 84.1% of women from sites in both countries were anemic with a hemoglobin level of 3–11 g/dL. Pakistani women were more likely to have moderate or severe anemia compared to Indian women. In the Pakistani site, the rate of severe anemia defined by a hemoglobin level of < 7 g/dL was substantially higher compared to the rate in the Indian sites (6.9% vs 0.2%) [16]. The socioeconomic status data for the Pakistani site shows a mean score that falls near the middle among the GN sites [17]. The percentage of women with a short IDI was also very different between the Pakistani site and the other sites. Twenty percent of the women in the Pakistani site had an IDI of less than 18 months compared to 7.6% in the other sites combined [18].

With regards to delivery care, the Pakistani site differed significantly from the Indian sites but not as much from some of the other sites. In the Pakistani site, approximately 40% of deliveries took place at home or in a location outside a health care facility. In the Indian sites, less than 5% of the deliveries occurred outside a facility. In Guatemala, just under 50% of deliveries occurred outside a health facility (Table 4). A similar variation occurs for type of delivery attendant. In the Pakistani site, 52% of deliveries were conducted by non-skilled personnel, whereas in the Indian sites, only 4% were conducted by non-skilled personnel. Rates of non-skilled attendance in the other sites fell in between. The percentage of cesarean deliveries ranged from 1.2% in the DRC and 1.6% in the other African sites, to 24.5% in the Guatemalan site. The cesarean delivery rates in the Pakistani and Indian sites fell between these rates, at 11.3% and 20.1% respectively (Table 4).

**Table 4:** Delivery characteristics for women for each of the sites and for all the sites combined except Pakistan

Variable	Pakistan	India	Kenya and Zambia	DRC	Guatemala	Total except Pakistan
Deliveries, N	91,076	205,203	136,567	31,604	82,902	456,276
Delivery location, N (%)	90,982	205,089	136,527	31,574	82,892	456,082
Hospital	29,890 (32.9)	138,667 (67.6)	25,943 (19.0)	2774 (8.8)	41,275 (49.8)	208,659 (45.8)
Clinic	24,430 (26.9)	57,820 (28.2)	63,463 (46.5)	22,084 (69.9)	1803 (2.2)	145,170 (31.8)
Home/other	36,662 (40.3)	8602 (4.2)	47,121 (34.5)	6716 (21.3)	39,814 (48.0)	102,253 (22.4)
Delivery attendant, N (%)	90,961	205,124	136,522	31,571	82,892	456,109
Physician	24,622 (27.1)	123,425 (60.2)	3422 (2.5)	394 (1.2)	43,986 (53.1)	171,227 (37.5)
Nurse/Midwife/HW	19,471 (21.4)	74,243 (36.2)	86,600 (63.4)	21,625 (68.5)	964 (1.2)	183,432 (40.2)
TBA	39,816 (43.8)	3252 (1.6)	31,468 (23.0)	7684 (24.3)	37,560 (45.3)	79,964 (17.5)
Family/other	7052 (7.8)	4204 (2.0)	15,032 (11.0)	1868 (5.9)	382 (0.5)	21,486 (4.7)
Delivery mode, N (%)	91,005	205,126	136,527	31,572	82,889	456,114
Vaginal	77,712 (85.4)	162,505 (79.2)	133,479 (97.8)	31,123 (98.6)	62,551 (75.5)	389,658 (85.4)
Vaginal assisted	3012 (3.3)	1418 (0.7)	848 (0.6)	83 (0.3)	33 (0.0)	2382 (0.5)
Cesarean delivery	10,281 (11.3)	41,203 (20.1)	2200 (1.6)	366 (1.2)	20,305 (24.5)	64,074 (14.0)
Attendant used new gloves, N (%)	49,178	120,819	65,625	–	33,339	219,783
Yes	37,651 (76.6)	117,740 (97.5)	64,607 (98.4)	–	32,884 (98.6)	215,231 (97.9)
No	11,527 (23.4)	3079 (2.5)	1018 (1.6)	–	455 (1.4)	4552 (2.1)
Clean razor to cut cord, N (%)	49,181	120,141	65,632	–	30,251	216,024
Yes	48,114 (97.8)	119,281 (99.3)	64,995 (99.0)	–	19,668 (65.0)	203,944 (94.4)
No	1067 (2.2)	860 (0.7)	637 (1.0)	–	10,583 (35.0)	12,080 (5.6)
Fetal heart rate taken, N (%)	49,152	120,116	65,532	–	33,462	219,110
Yes	20,789 (42.3)	116,023 (96.6)	42,140 (64.3)	–	20,965 (62.7)	179,128 (81.8)
No	28,363 (57.7)	4093 (3.4)	23,392 (35.7)	–	12,497 (37.3)	39,982 (18.2)

Characteristics compared between the Pakistan site and all other sites combined using chi-square tests. All p values were < 0.0001

While Pakistani women were as likely to have a facility-based delivery as their counterparts in the Guatemalan and Kenyan and Zambian sites, the quality of delivery care appeared poorer. As previously mentioned, unlike women in the Kenyan and Zambian sites, Pakistani women were less likely to be delivered by a skilled birth attendant. Furthermore, in Pakistan, only three-quarters of delivery attendants used gloves during the delivery compared to more than 97% in all other sites. Similarly, the fetal heart rate was measured in less than half of the deliveries in the Pakistani site, compared to more than 80% in the other sites combined (Table 4).

Neonatal care also seemed to be substantially worse in the Pakistani site compared to the combined rates at the other sites, as evidenced by much lower rates of skin to skin placement (7.9% vs. 61.4%), medicinal cord care (6.6% vs. 42.3%), immunization (3.8% vs. 49.7%), and early initiation of breastfeeding (15.4% vs. 79.9%) (Table 5).

**Table 5:** Fetal/neonatal characteristics and newborn care for each of the sites and for all the sites combined except Pakistan

Variable	Pakistan	India	Kenya and Zambia	DRC	Guatemala	Total except Pakistan
Births, N	92,166	206,816	138,146	32,173	83,481	460,616
Birth weight, N (%)	91,535	206,342	137,936	32,118	83,354	459,750
< 1000 g	819 (0.9)	1,560 (0.8)	330 (0.2)	196 (0.6)	361 (0.4)	2447 (0.5)
100–1499 g	1770 (1.9)	2,489 (1.2)	719 (0.5)	414 (1.3)	557 (0.7)	4179 (0.9)
1500–2499 g	16,580 (18.1)	32,576 (15.8)	6278 (4.6)	3592 (11.2)	12,255 (14.7)	54,701 (11.9)
≥ 2500 g	72,366 (79.1)	169,717 (82.3)	130,609 (94.7)	27,916 (86.9)	70,181 (84.2)	398,423 (86.7)
Preterm, N (%)	88,120	202,176	132,773	32,047	81,492	448,488
Yes	18,104 (20.5)	22,371 (11.1)	16,458 (12.4)	6733 (21.0)	8911 (10.9)	54,473 (12.1)
No	70,016 (79.5)	179,805 (88.9)	116,315 (87.6)	25,314 (79.0)	72,581 (89.1)	394,015 (87.9)
Neonatal care						
Placed on chest/skin to skin care, N (%)	89,678	201,434	135,921	30,818	81,282	449,455
Yes	7066 (7.9)	118,069 (58.6)	100,824 (74.2)	23,430 (76.0)	33,489 (41.2)	275,812 (61.4)
No	82,612 (92.1)	83,365 (41.4)	35,097 (25.8)	7,388 (24.0)	47,793 (58.8)	173,643 (38.6)
Cord care, N (%)	61,051	140,630	99,119	32,080	69,654	341,483
Yes	4047 (6.6)	49,760 (35.4)	24,772 (25.0)	2067 (6.4)	67,896 (97.5)	144,495 (42.3)
No	57,004 (93.4)	90,870 (64.6)	74,347 (75.0)	30,013 (93.6)	1758 (2.5)	196,988 (57.7)
Immunization, N (%)	19,087	56,110	27,676	–	20,031	103,817
Yes	726 (3.8)	32,357 (57.7)	8026 (29.0)	–	11,179 (55.8)	51,562 (49.7)
No	18,361 (96.2)	23,753 (42.3)	19,650 (71.0)	–	8852 (44.2)	52,255 (50.3)
Breast feeding within 1 h, N (%)	89,644	201,202	136,375	30,798	81,427	449,802
Yes	13,833 (15.4)	162,732 (80.9)	113,480 (83.2)	29,844 (96.9)	53,176 (65.3)	359,232 (79.9)
No	75,811 (84.6)	38,470 (19.1)	22,895 (16.8)	954 (3.1)	28,251 (34.7)	90,570 (20.1)

Characteristics compared between the Pakistan site and all other sites combined using chi-square tests. All p values were < 0.0001

The Pakistani site also had the highest proportion of children born with low birthweight (LBW), with 20.9% of newborns weighing below 2500 g. In comparison, the proportion of LBW babies was 17.8% in the Indian sites, 5.3% in the combined Kenyan and Zambian sites, 13.1% in the DRC, and 15.8% in Guatemala (Table 5). The rate of preterm births in Pakistan was 20.5%. In contrast, all other sites with the exception of the DRC had rates closer to 12%.

We evaluated the fetal and neonatal mortality rates amongst babies in each of the birthweight groups (Table 6). In most weight ranges, Pakistani babies had significantly higher rates of stillbirth and neonatal mortality than those in the other sites combined with differences increasing as the birthweight increased. Babies born weighing ≥ 2500 g are an important group to consider because such a large percentage of babies are in this group and mortality should be low. Pakistani newborns ≥ 2500 g had two to three times greater risk of neonatal mortality and stillbirth compared to the other sites combined.

**Table 6:** Neonatal mortality and stillbirth rates by birth weight group in each site and for all the sites combined except Pakistan

Variable	Pakistan	India	Kenya and Zambia	DRC	Guatemala	Total except Pakistan	Pakistan vs. Total except Pakistan RR (95% CI)
Mortality rates for births < 1000 g							
Births < 1000 g, N (%)	819 (0.9)	1560 (0.8)	330 (0.2)	196 (0.6)	361 (0.4)	2447 (0.5)	
Stillbirth, n/N (rate/1000)	635/817 (777.2)	1183/1560 (758.3)	234/330 (709.1)	137/196 (699.0)	215/361 (595.6)	1769/2447 (722.9)	1.10 (1.04, 1.16)
28-day neonatal mortality, n/N (rate/1,000)	164/179 (916.2)	332/377 (880.6)	76/94 (808.5)	49/59 (830.5)	136/146 (931.5)	593/676 (877.2)	1.04 (0.99, 1.10)
Mortality rates for births 1000–1499 g							
Births 1000–1499 g, N (%)	1770 (1.9)	2489 (1.2)	719 (0.5)	414 (1.3)	557 (0.7)	4179 (0.9)	
Stillbirth, n/N (rate/1,000)	838/1,770 (473.4)	998/2489 (401.0)	348/719 (484.0)	167/414 (403.4)	172/557 (308.8)	1685/4179 (403.2)	1.16 (1.04, 1.29)
28-day neonatal mortality, n/N (rate/1000)	559/927 (603.0)	774/1484 (521.6)	199/367 (542.2)	138/247 (558.7)	214/383 (558.7)	1325/2481 (534.1)	1.12 (1.04, 1.22)
Mortality rates for births 1500–2499 g							
Births 1500–2499 g, N (%)	16,580 (18.1)	32,576 (15.8)	6278 (4.6)	3592 (11.2)	12,255 (14.7)	54,701 (11.9)	
Stillbirth, n/N (rate/1000)	1292/16,580 (77.9)	1456/32,576 (44.7)	668/6278 (106.4)	330/3592 (91.9)	450/12,255 (36.7)	2904/54,701 (53.1)	1.20 (1.04, 1.38)
28-day neonatal mortality, n/N (rate/1000)	1591/15,195 (104.7)	1654/31,079 (53.2)	486/5561 (87.4)	227/3260 (69.6)	666/11,783 (56.5)	3033/51,683 (58.7)	1.63 (1.48, 1.80)
Mortality rates for births ≥ 2500 g							
Births > 2500 g, N (%)	72,366 (79.1)	169,717 (82.3)	130,609 (94.7)	27,916 (86.9)	70,181 (84.2)	398,423 (86.7)	
Stillbirth, n/N (rate/1000)	1630/72,366 (22.5)	1023/169,717 (6.0)	1416/130,609 (10.8)	594/27,916 (21.3)	673/70,181 (9.6)	3706/398,423 (9.3)	2.24 (1.90, 2.65)
28-day neonatal mortality, n/N (rate/1000)	1942/70,125 (27.7)	1721/168,570 (10.2)	1193/128,581 (9.3)	325/27,300 (11.9)	908/69,349 (13.1)	4147/393,800 (10.5)	2.60 (2.34, 2.87)

Table 7 shows the MMR, stillbirth and NMR for community and facility births for the Pakistani site and for the other sites combined. For facility births in Pakistan, the MMR, stillbirth and NMR were more than twice that for the other sites, while the differences between Pakistan and the other sites for community births were less consistent.

**Table 7:** Mortality rates by delivery location for Pakistan compared to the other sites and all the other sites excluding Pakistan

Variable	Pakistan	India	Kenya and Zambia	DRC	Guatemala	Total except Pakistan	Pakistan vs. Total except Pakistan RR (95% CI)
Community mortality rates							
Community births, N (%)	36,924 (40.1)	8641 (4.2)	47,568 (34.4)	6828 (21.2)	39,952 (47.9)	102,989 (22.4)	
MMR < 42 days, n/N (rate/100,000 deliveries)	65/36,354 (179)	8/8594 (93)	39/46,954 (83)	23/6710 (343)	36/39,724 (91)	106/101,982 (104)	1.72 (1.30, 2.28)
Stillbirth, n/N (rate/1000)	1948/36,922 (52.8)	782/8641 (90.5)	1023/47,567 (21.5)	328/6828 (48.0)	705/39,952 (17.6)	2838/102,988 (27.6)	0.76 (0.60, 0.97)
28-day neonatal mortality, n/N (rate/1000)	1531/34,669 (44.2)	318/7854 (40.5)	689/46,380 (14.9)	183/6495 (28.2)	805/39,169 (20.6)	1995/99,898 (20.0)	1.72 (1.49, 1.99)
Facility mortality rates							
Facility births, N (%)	55,147 (59.9)	198,057 (95.8)	90,538 (65.6)	25,315 (78.8)	43,519 (52.1)	357,429 (77.6)	
Maternal deaths < 42 days, n/N (rate/100,000 deliveries)	146/53,893 (271)	155/196,347 (79)	65/88,915 (73)	45/24,835 (181)	33/42,965 (77)	298/353,062 (84)	3.15 (2.57, 3.86)
Stillbirth, n/N (rate/1000)	2974/55,146 (53.9)	4236/198,055 (21.4)	1783/90,538 (19.7)	921/25,315 (36.4)	905/43,519 (20.8)	7845/357,427 (21.9)	2.34 (2.07, 2.64)
28-day neonatal mortality, n/N (rate/1000)	2735/51,761 (52.8)	4170/193,652 (21.5)	1278/88,251 (14.5)	558/24,375 (22.9)	1131/42,509 (26.6)	7137/348,787 (20.5)	2.48 (2.24, 2.75)

## Discussion

The pregnancy outcomes of maternal mortality, stillbirth and neonatal mortality in Pakistan are among the worst in the world, and certainly worse than the values for the other sites combined. In the last five years, there has been some improvement in stillbirth rates in the Pakistani site but there is no evidence of improvement in the MMR or the NMR. In this paper, we explored potential reasons for the very high mortality in Thatta, Pakistan by comparing various factors in the Pakistani site to those in the other GN sites.

Among the maternal characteristics in the Pakistani site compared to the other sites, the one that stands out the most is lack of formal education. Nearly 83% of Pakistani women had no formal education compared to only 12% in the other sites combined. National reports by UNICEF and the PDHS have highlighted that low educational level is a key factor associated with poor access to critical maternal health services as well as poorer maternal and child health outcomes [7, 19].

Another maternal characteristic that stands out as an important difference between the Pakistani and the other sites is the inter-delivery interval (IDI), which was shorter among women in the Pakistan site versus women in the other sites. Twenty percent of the deliveries in the Pakistani site had an IDI of less than 18 months compared to 7.6% in the other sites. This factor could be linked to a higher risk for adverse maternal, fetal, and newborn outcomes, depending on IDI’s degree of brevity. For example, the PDHS highlighted that infant mortality rates are higher among children born fewer than two years after a previous birth than among children born two or more years after a previous birth [8].

The high percentage of pregnant women with anemia and especially severe anemia is another distinctive characteristic of the Pakistani site. For example, about 81% of women in the Pakistani site were anemic with a hemoglobin level of < 11 g/dL, similar to the rate in the Indian site [20]. However, 6.9% of Pakistani women had a hemoglobin level of < 7 g/dL compared to 0.2% in India [16]. In a recent publication, anemia has been associated with a doubling of the maternal mortality ratio [21].

Compared to the women from the other sites combined, women in Pakistan had lower a BMI, although the percent of women with a low BMI was similar to women in the Indian sites. Low BMI, especially when combined with inadequate weight gain in pregnancy, is associated with an adverse effect on birth weight and an increased risk for having small for gestational age and preterm births [22, 23].

Finally, among the maternal characteristics that may help to explain part of the difference in the mortality outcomes, is the large percentage of women at high parity in Thatta. The PDHS found that increased parity was a risk factor for decreased access to and use of quality ANC services, health facility delivery, and postnatal care for women in Pakistan [8].

There are a number of factors related to ANC that were different between the Pakistani and the other sites, including the number of ANC visits, the provision of iron and vitamins and the use of tetanus toxoid. These disparities could be an indicator of poor functioning of Pakistan’s LHW program or low availability of LHWs in the Thatta site. For delivery characteristics, the Pakistani site had fewer facility deliveries and physician-attended deliveries compared to the other sites combined; however, these delivery rates varied broadly among the other sites with some sites having lower observed rates than the Pakistani site. Delivery attendants in the Pakistani site were less likely to monitor the fetal heart rate prior to delivery or to use gloves for delivery. Neonatal care also differed between the Pakistani and the other sites. In the Pakistani site compared to other sites combined, there was less medicinal cord care, neonatal immunization, skin-to-skin placement, and early-initiation of breastfeeding. Higher rates were observed at each of the other sites for all neonatal care except for medicinal cord care in the DRC. Thus, at every level of care, there is evidence that the human and material resources available to pregnant women and their newborns in the Pakistani site were likely insufficient. We also examined the impact on mortality of delivering in a facility in the Pakistani site to delivering in a facility in the other sites and similarly, we compared community and facility deliveries. Regardless of where the deliveries took place, the outcomes were generally worse in the Pakistani compared to the other sites.

We also compared the newborns at the Pakistani site compared to the other sites combined. The Pakistani site had a higher proportion of preterm and LBW infants and the survival of fetuses and newborns in each birthweight group was lower in Pakistan compared to the other sites combined. The potential reasons could be an inadequate community-based health care delivery system, especially in rural areas, to address the needs of pregnant women and newborns.

Factors studied that did not seem likely to explain the differences in maternal, fetal and newborn outcomes in Pakistan compared to the other sites included a measure of socioeconomic status (SES). The SES index score for the Pakistani site was approximately the average among the sites, and therefore it does not seem that housing conditions and household assets could be the predominant explanation for the worse outcomes in Thatta [17]. The proportion of cesarean deliveries in the Pakistani site was 11.3%, an acceptable rate per the WHO, in the middle among the sites, and likely does not account for the differences in pregnancy outcomes. Also, because the Pakistani site had a much lower rate of teen pregnancy, the maternal age differences observed between the Pakistani and other sites are not likely to account for much of the mortality differences. In another study comparing progress to the MDGs among high-burden countries, authors found that there were a number of factors, including state governance, conflict and women’s empowerment indicators that were significantly worse in Muslim-majority countries, such as Pakistan, compared to non-Muslim-majority countries [24]. These factors, such as women’s empowerment and conflict, are also strongly associated with high burden of maternal and newborn mortality and likely explanative, but beyond the scope of our study.

This study had its strengths and weaknesses. Among its strengths were the large sample size and that it included data from seven sites in six countries, and the fact that the data were prospectively collected using standard methods across the sites. We are reasonably confident that few subjects were missed, but in a study of more than 500,000 subjects, it is likely that there were some errors made in data collection and coding. However, the data is relatively consistent from year to year in all sites and small changes would not have changed the overall picture presented. The data from the Pakistani site are generally similar to data collected in the PDHS and other surveys. Among weaknesses, multivariate regression analyses were beyond the scope of this paper as we did not have data to evaluate additional contextual factors that may account for poor outcomes in the Pakistan site.

## Conclusions

There are large differences in pregnancy outcomes, medical care, and numerous maternal and neonatal characteristics between the Pakistani and other GN sites. That the outcomes in Pakistan are worse is clear; however, it is less obvious which of the maternal and neonatal factors, as well as the various neonatal, labor, and delivery practices, are responsible for the mortality differences. The Pakistani site has very high rates of preterm delivery and LBW, so the prognosis for babies born at this site is poorer than in the other sites. Furthermore, within each birthweight group—even above 2500 g, the mortality in Pakistan is higher. Indian and Pakistani women have many similar characteristics in terms of nutritional status, diet, and BMI, so it is challenging to attribute the large differences in outcome to those features alone, although the rate of severe anemia in Pakistan may offer some insight. Probably more important, are the very low educational levels, the short IDIs, and high parity found among the Pakistani women. Compounding these issues is the suboptimal medical care in the Thatta site at every level examined compared to the other sites. We hypothesize that the poor mortality results are due to a combination of the aforementioned factors.

Ascribing responsibility among the factors studied for Pakistan’s increased stillbirth rate, maternal mortality ratio, and neonatal mortality rate will be difficult. Probably more important is the question of how to improve the outcomes described here. What seems obvious to us is that there should be no reason Pakistani maternal, fetal and neonatal outcomes cannot be improved to be at least similar to those in other south Asian countries. The specific interventions required are multiple, likely complex, and probably need to be introduced together. Improving the educational levels and nutritional status of pregnant women would be a good place to start. Achieving substantial improvements in the care provided at all levels of the health care system for pregnant women and children seems to be crucial.

It will be complicated and require additional resources and a lot of effort; however, it does not seem impossible to achieve substantial improvements in maternal, fetal and neonatal health in Pakistan. Without addressing the factors noted above, including substantially strengthening the health care system, and probably many other factors not evaluated in this paper, it is unlikely that Pakistan will reach the ambitious mortality goals set for each country by the SDGs 2015–2030.

To summarize, it is not difficult to understand why the Pakistani pregnancy outcomes are so much worse than those in the other GN sites, considering that Thatta’s reproductive-aged women are largely poorly educated, undernourished, anemic, and deliver a high percentage of preterm and low birthweight babies in a setting of often inadequate maternal and newborn care. A multipronged approach to addressing the issues highlighted in this paper has the potential to achieve substantial improvements in Pakistan’s pregnancy outcomes.

## Data Availability

Data from the study will be available at the NICHD data repository (N-DASH): https://dash.nichd.nih.gov/.

## Abbreviations

MNHR: Maternal Newborn Health Registry
MMR: Maternal mortality ratios
NMR: Neonatal mortality rates
MDGs: Millennium development goals
PDHS: Pakistan Demographic Health Survey
MNCH: National Maternal, Neonatal, and Child Health Programme
LHWs: Lady health workers
SDGs: Sustainable development goals
DRC: Democratic Republic of the Congo
NICHD: *Eunice Kennedy Shriver* National Institute of Child Health and Human Development
GN: Global Network for Women and Children’s Health Research
ANC: Antenatal care
SES: Socioeconomic status
IDI: Inter-delivery interval
BMI: Body mass index
